# Ezrin Ubiquitylation by the E3 Ubiquitin Ligase, WWP1, and Consequent Regulation of Hepatocyte Growth Factor Receptor Activity

**DOI:** 10.1371/journal.pone.0037490

**Published:** 2012-05-22

**Authors:** Rania F. Zaarour, Dafne Chirivino, Laurence Del Maestro, Laurent Daviet, Azeddine Atfi, Daniel Louvard, Monique Arpin

**Affiliations:** 1 Institut Curie-Unité Mixte de Recherche Centre National de la Recherche Scientifique, Paris, France; 2 Hybrigenics, Paris, France; 3 INSERM U, Hôpital St-Antoine, Paris, France; Yale Medical School, United States of America

## Abstract

The membrane cytoskeleton linker ezrin participates in several functions downstream of the receptor Met in response to Hepatocyte Growth Factor (HGF) stimulation. Here we report a novel interaction of ezrin with a HECT E3 ubiquitin ligase, WWP1/Aip5/Tiul1, a potential oncogene that undergoes genomic amplification and overexpression in human breast and prostate cancers. We show that ezrin binds to the WW domains of WWP1 via the consensus motif PPVY^477^ present in ezrin’s C-terminus. This association results in the ubiquitylation of ezrin, a process that requires an intact PPVY^477^ motif. Interestingly ezrin ubiquitylation does not target the protein for degradation by the proteasome. We find that ezrin ubiquitylation by WWP1 in epithelial cells leads to the upregulation of Met level in absence of HGF stimulation and increases the response of Met to HGF stimulation as measured by the ability of the cells to heal a wound. Interestingly this effect requires ubiquitylated ezrin since it can be rescued, after depletion of endogenous ezrin, by wild type ezrin but not by a mutant of ezrin that cannot be ubiquitylated. Taken together our data reveal a new role for ezrin in Met receptor stability and activity through its association with the E3 ubiquitin ligase WWP1. Given the role of Met in cell proliferation and tumorigenesis, our results may provide a mechanistic basis for understanding the role of ezrin in tumor progression.

## Introduction

The activation of the Met receptor by its ligand, the Hepatocyte Growth Factor (HGF), elicits complex biological responses in epithelial cells. HGF induces proliferative and anti-apoptotic signals. It also triggers cell scattering and motility and promotes morphogenic programs when cells are cultured in three-dimensional matrix [1].

It has been shown that the membrane-cytoskeleton linker, ezrin, participates in several events induced by Met activation. Ezrin belongs to the ERM (Ezrin, Radixin, Moesin) protein family. The activity of these proteins is negatively regulated by an intramolecular interaction between their N-terminal and C-terminal domains that masks their membrane and actin cytoskeleton binding sites [2]. Activation of the proteins requires conformational changes triggered by their sequential binding to PIP2 followed by phosphorylation of a conserved threonine residue in their COOH-terminus [3]. Following HGF stimulation of epithelial cells, ezrin is phosphorylated at specific tyrosine residues involved in various signalling pathways [4], [5]. We have shown, in a tubulogenesis assay, that phosphorylation of ezrin at tyrosine 353 signals cell survival through the PI3-K pathway [5] whereas its phosphorylation at tyrosine 145 controls cell proliferation [6]. The association of ERM (Ezrin, Radixin, Moesin) proteins with CD44v6, a coreceptor of Met, is necessary for HGF-mediated activation of Ras by the guanine nucleotide exchange factor Sos [7]. Moreover, ezrin is involved in several HGF-induced functions that require actin cytoskeleton remodeling such as cell morphogenesis and motility [4], [7]–[9]. In particular, ezrin phosphorylation at tyrosine 477 is required for HGF-induced cell scattering through the recruitment and activation of the Fes kinase [9].

The remodeling of the actin cytoskeleton and adhesion complexes triggered by HGF are mediated by non receptor tyrosine kinases from the Src family [4], [6], [9]. Because ezrin is a substrate of Src family kinases [6], [10] we performed a modified two-hybrid screen to identify the proteins interacting with ezrin phosphorylated by Src family kinase. We identified an interaction of ezrin with WWP1/AIP5/Tiul1 a type E3 ubiquitin ligase that belongs to the HECT family (Homologous to the E6-associated protein C terminus) [11].

Through ubiquitylation of different targets, WWP1 has been implicated in the regulation of cell growth and apoptosis. WWP1 was found to target transcription factors including p53 [12], the Krüppel-like factors KLF2 [13], KLF5 [14], p63 [15] and Runx2 [16]. WWP1 has also a regulatory role in receptor signalling. A negative regulation of TGF-β signalling by WWP1 has been reported [17], [18]. Through its interaction with Smad7, WWP1 causes ubiquitylation and degradation of the TGF-β receptor type 1 [18]. WWP1 can also interact with TGIF to induce the degradation of Smad 2 [17]. Recently, it was shown that WWP1 upregulates the activity of ErbB2 and EGF receptors [19]. This occurs through the interaction of WWP1 with ring finger protein 11 (RNF11), a negative regulator of these receptors.

Here we report a novel mechanism by which ezrin regulates the level and the activity of Met through its interaction with WWP1. We found that the interaction between WWP1 and ezrin is mediated by the WW domains of WWP1 and the PPVY^477^ motif present in ezrin. We observed that ezrin is ubiquitylated by WWP1 however this ubiquitylation does not target ezrin for degradation. Rather, the interaction of ezrin with WWP1 increases the levels of Met in absence of HGF stimulation. This effect requires ezrin since depletion of ezrin in cell lines stably expressing WWP1 abolishes the stabilization of Met. Moreover, we show that cells expressing WWP1 display an increase in wound healing response when stimulated with HGF and that this response requires ubiquitylated ezrin. Altogether our data suggest that Met stability is regulated via a pathway that requires the joint active function of ezrin and WWP1.

## Materials and Methods

### Reagents and Antibodies

The following antibodies were used: affinity-purified rabbit polyclonal anti-vesicular stomatitis virus glycoprotein epitope (VSVG) and anti-ezrin antibodies [20]; mouse monoclonal anti-Myc (clone 9E10); mouse monoclonal anti- tubulin, Sigma-Aldrich; Rat monoclonal anti E-cadherin (Zymed); mouse monoclonal anti-Flag M2 (Sigma-Aldrich); mouse monoclonal anti-Met (clone 25F2; Cell Signaling); polyclonal anti-WWP1 antibody was generated against a peptide of WWP1 aa 163–176 C-NGDALHENGEPSAR. Peptide and rabbit immunization were performed by Covalab. Affinity immunopurification was performed against His-WWP1 (aa 1–413). Horseradish peroxidase–conjugated goat anti-rabbit, anti-mouse, anti-rat secondary antibodies, Cy3- and Alexa 488–conjugated goat anti-rabbit, anti-mouse, anti-Rat 546 secondary antibodies were from Jackson ImmunoResearch Laboratories. Rhodamine-phalloidin and Alexa Fluor 350 phalloidin were from Molecular Probes.

### Plasmids and cDNA Constructs

Ezrin plasmids for eukaryotic expression and GST-tagged plasmids were described previously [21], [22]. Point mutations were generated using the QuickChange mutagenesis kit (Stratagene, La Jolla, CA). Flag-tagged WWP1 and WWP1 C890A in CMV 7.1 plasmid were described previously [17]. GFP-WWP1 was generated by PCR of WWP1 and inserted into pEGFP C2. His-tagged WWP1 (aa 1–413) was generated by PCR and inserted into the FseI and AscI sites of pET28 (pET plasmids were a gift from Dr. A. Gautreau.). PC tagged WWP1 was cloned in pCDNA5/FRT/V5-His modified as described previously [23]. GST-fusions of various WWP1 domains were performed as follows: GST-WWP1 full length was cloned into pGEX 4T-1 into EcoRI 5′ and NotI 3′; GST-WW domain 1 (aa 350–381), WW domain 2 (aa 382–412), WW domain 3 (aa 456–488), WW domain 4 (aa 496–528), were cloned by PCR of the listed region into pGEX 4T-1 using EcoRI 5′ and Not1 3′. The plasmids pcDNA3 encoding VSVG-tagged wild type ubiquitin, ubiquitin KO mutated on all lysines or mutated on all but one of the lysine residues involved in the polymerization of ubiquitin molecules were a kind gift from Dr. Christel Brou [24].

### RNA Interference

For depletion of WWP1, 293T cells were transfected with the ON-target plus SMART pool from Thermo Scientific Dharmacon (UK) at the concentration of 10 nM, using RNAiMAX (Invitrogen). Cells were analyzed at different times after transfection and complete depletion was achieved 4 days after transfection.

### Cell Culture and Transfection

293T cells and LLC-PK1 cells (CCL 101 American Type Culture Collection, Molsheim) were grown in Dulbecco’s modified Eagle’s medium (Invitrogen) supplemented with 10% fetal bovine serum and maintained in 10% CO_2_ at 37°C. 293T cells were transiently transfected by the calcium phosphate method and analyzed 24 or 48 h after transfection. Pools of LLC-PK1 cells stably expressing GFP or GFP-WWP1 were obtained by selection with G418 (0.7 g/l) after cell transfection by electroporation with the corresponding plasmids. The stable Flp-In 293 cell lines expressing WWP1 were obtained according to the manufacturer’s protocols (Invitrogen) and grown in presence of 200 µg/ml Hygromycin. The stable Flp-In™ T-REx™ 293 (Invitrogen) cell line expressing PC tagged WWP1 was obtained as described previously [23] and grown in presence of 200 µg/ml Hygromycin.

### Immunoprecipitation and GST Pull-down Experiments

Cells transfected with the appropriate plasmid(s), were washed with cold PBS followed by lysis in RIPA buffer (50 mM Hepes pH 7.5, 10 mM EDTA, 150 mM NaCl, 1% Nonidet P-40, 0.1% SDS; 0.5% deoxycholate) 1 mM orthovanadate when indicated, plus protease inhibitors cocktail (Sigma). The cell lysates were spun at 20.000×*g* for 20 min; the cleared lysates were incubated for two hours at 4°C with the indicated antibody for immunoprecipitation together with 10 µl protein-G beads (Perbio), or with various GST-tagged proteins bound to glutathione Sepharose 4B (GE Healthcare) for pull-down experiments. Beads were then washed 4×500 µl with RIPA buffer. For ubiquitylation analysis, samples were ran on 3–8% gradient NuPAGE Tris-Acetate gels (Invitrogen).

### Membrane Cytosol Fractionation

Cytosol/membrane fractionation was performed as described previously [3]. In brief, cells were mechanically disrupted using a cell cracker in PBS containing a cocktail of protease inhibitors (Sigma). The homogenates were clarified by centrifugation at 600×*g*. The resultant supernatant was spun at 100,000×*g* for 30 min to obtain the membrane fraction. Supernatant and membrane pellets were analyzed by SDS-PAGE and immunoblotting.

### In vitro and in vivo Ubiquitylatio

For in vitro ubiquitylation assay, 293T cells were transfected with the plasmid encoding GFP-WWP1 and the protein was purified using the GFP-Trap method according to manufacturer’s recommendation (Chromotek; Planegg-Martinsried). GST-ezrin was purified as described above and dialyzed against the ubiquitylation buffer. The in vitro ubiquitylation reactions were carried out at 30°C for 1 h in 50 µl of ubiquitylation reaction buffer (25 mM Tris-HCl at pH 7.6, 5 mM MgCl_2_, 0.1 M NaCl, 1 mM DTT, 2 mM ATP supplemented with the protease inhibitor cocktail, and the phosphatase inhibitor cocktail II from Sigma) containing the following components: 100 ng of E1 (BostonBiochem, Cambridge, MA), 150 ng of UbcH5a (BostonBiochem), 5 µg of Myc-ubiquitin (BostonBiochem), ∼1 µg GFP-WWP1 coupled to beads and 1,5 µg purified GST-ezrin. The reactions were terminated by adding the Laemmli buffer to the beads.

For the in vivo ubiquitylation experiments in which denatured extracts were used, cells expressing Flag-WWP1, ezrin-VSVG and Myc-His-ubiquitin were lysed in RIPA buffer without EDTA, the extracts were adjusted to 1% SDS and boiled for 2 min. Denatured extracts were then put on ice for 10 min and diluted with cold RIPA buffer without EDTA to adjust SDS concentration to 0.5%. For non denaturing conditions, cells were lysed in RIPA buffer without EDTA. The lysates were incubated with TALON beads precharged with Co^2+^ (Clontech, Saint-Germain en Laye, France). After 1 h incubation, beads were washed and proteins were solubilized in Laemmli buffer.

### Pulse Chase Experiments

Metabolic labeling was performed with 250 µCi/ml ^35^S-labeled Met and Cys from Redivue Promix (Amersham Biosciences). Flp-In™ T-REx™ 293 cells stably expressing PC tagged WWP1 or transfected with the empty plasmid were grown in 6 cm dish, labeled for 15 min and chased for the indicated time in standard DMEM containing 10% FBS. After immunoprecipitation and SDS-PAGE, signals were quantified using a STORM 860 PhosphorImager and ImageQuant software (Amersham Biosciences).

### Immunofluorescence

Cells were fixed with PFA (4%) and processed for immunofluorescence. Images were taken with a 90i upright Microscope Nikon Microsystems equipped with PIFOC Objective stepper and a 100X/1.4 N.A. Plan Apochromat objective. Slices were acquired along the Z axis every 0.2 µm. Deconvolution was done by the Metamorph module (Roper Scientific) using the Meinel algorithm.

### Met Stability and Activity

Met level was measured in LLC-PK1 cells stably expressing GFP or GFP-WWP1 after 5 hours starvation in DMEM supplemented with 1% FBS. For ezrin depletion, LLC-PK1 cells expressing GFP-WWP1 were electroporated with plasmids coding for scrambled shRNA or shRNA targeting porcine ezrin and for the reporter m-cherry [9]. The Met levels were analyzed four days after transfection as described above. Significance was tested using a paired t-test (SigmaStat).

For the wound healing assay, LLC-PK1 cells expressing GFP or GFP-WWP1 were plated onto ibiTreat dishes (IBIDI) and starved for 5 hours in DMEM supplemented with 1% FBS. The cell monolayer was wounded with a 10 µl tip, and when indicated HGF (120 ng/ml) was added to the medium. Cells were imaged for 16 hours at 37°C and 5% CO_2_ with an Eclipse Ti Inverted Microscope (Nikon) equipped with a 20x/0.45 NA Plan Fluor ELWD objective, a CoolsnapHQ2 camera (Roper) and The Cube and The Box (Life Imaging Services). For each condition, single cell speed was measured at 13 different positions along the wound with Metamorph software. Only cells expressing the indicated proteins and shRNA, detected through the expression of fluorescent reporters, were analyzed.

## Results

### Identification of the E3 Ubiquitin Ligase, WWP1, as an Ezrin Partner

**Figure 1:**
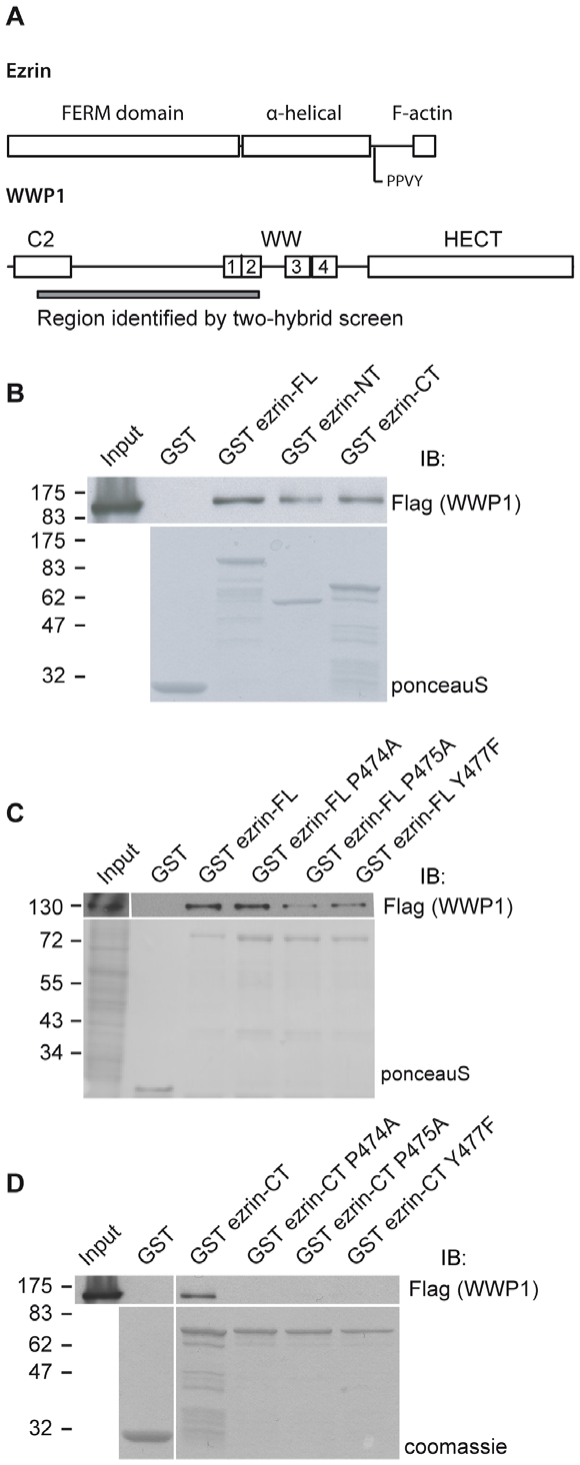
Ezrin interacts with the E3 ubiquitin ligase WWP1. (A) Schematic representation of the structural organization of ezrin and WWP1. (B) Lysates from 293T cells transfected with Flag-WWP1 were incubated with immobilized GST or GST fused to full length, N-ter or C-ter ezrin and the blot was performed with the anti-Flag antibody. Lower panel: the blot was stained with ponceau S to reveal the GST-tagged proteins. (C) An extract of 293T cells transfected with Flag-WWP1 was incubated with immobilized GST or GST fused to ezrin wild type or carrying mutations in the PPVY motif. Lower panel: Ponceau S staining of the blot. (D) Extract of cells transfected with Flag-WWP1 was incubated with immobilized GST or GST fused to the C-ter domain of ezrin either wild type or carrying the mutation in the PPVY motif. Lower panel: Coomassie staining of GST tagged proteins.

We have performed a two-hybrid screen with yeast transformed or not with the Lyn kinase to identify partners of ezrin phosphorylated by Src family kinases [9]. We used full-length ezrin and ezrin deleted of the last 52 amino acids as baits. Deleting the last 52 amino acids disrupts the strong intramolecular interaction between the N-terminal and C-terminal domains, an interaction that is likely to mask binding sites to ezrin partners [2]. WWP1 was identified in the screen performed with yeast transformed with the Lyn kinase and ezrin truncated for the last 52 amino acids but was neither found in the screen performed with full length ezrin as bait nor in the screen performed in the absence of the Lyn kinase. The region identified in the two-hybrid screen encompasses the C2 domain and the first two WW domains (aa 48–405) of WWP1 (Fig. 1A).

### Ezrin Interacts with WWP1 through its PPVY^477^ Motif

To confirm the interaction between WWP1 and ezrin we performed experiments to pull down Flag-WWP1 expressed in 293T cells with various GST-ezrin domains. We identified a specific interaction with GST-full length ezrin as well as with GST N-ter (aa 1–308) and GST C-ter (aa 308–585) domains of ezrin (Fig. 1B), indicating that there are two binding sites for WWP1 in ezrin, one in the C-terminus and the second in the N-terminus.

WWP1 contains four WW domains that belong to the class I family of WW domains, shown to bind to PPXY motifs [25]. The C-terminal domain of ezrin contains a PPXY motif (PPVY^477^), we therefore determined if the interaction of ezrin and WWP1 occurs through this. We generated GST-fusions of ezrin full length or C-terminal domain alone mutated at a specific residue in this motif (ezrin P474A, P475A or Y477F). Disrupting PPVY^477^ in full-length ezrin did not abolish the interaction of WWP1 with ezrin (Fig. 1C). However, disrupting PPVY^477^ in the C-terminal domain alone of ezrin completely abolished the interaction with WWP1 (Fig. 1D). These results support our above data showing that there is a binding site within the N-terminus of ezrin and show that the second binding site requires the C-terminal PPVY^477^ motif of ezrin.

**Figure 2:**
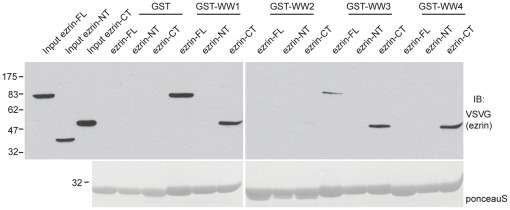
Mapping the interaction of ezrin with individual WW domain of WWP1. The lysates of 293T cells transfected with the indicated VSV-G tagged ezrin constructs were incubated with immobilized GST or GST fused to WW domains 1 to 4. Immunoblotting was performed with the anti-VSVG antibody. Lower panel: GST tagged proteins were revealed with ponceau S.

Separate WW domains from the same protein can have different specificity for protein ligands [26]. To test whether the WW domains of WWP1 have the same specificity towards ezrin, and to determine whether the recognition in the N-terminal domain of ezrin is mediated by a WW domain interaction, we performed pull down experiments using GST tagged WW domains 1 through 4 of WWP1 with various domains of VSVG-ezrin expressed in 293T cells. WW domains 1 and 3 were found to interact with full-length and with the C-ter domain of ezrin (Fig. 2). No interaction of WW domain 2 with either forms of the protein was found while WW domain 4 interacted only with the C-ter domain of ezrin. No interaction was detected between the N-ter domain of ezrin and any of the WW domains, indicating that the interacting site of WWP1 within the N-ter domain of ezrin is not WW domain dependent.

### Ezrin and WWP1 Colocalize at the Membrane

**Figure 3:**
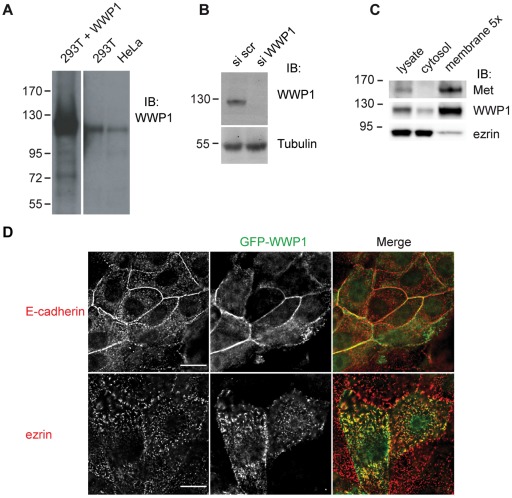
Subcellular localization of ezrin and WWP1. (A) Immunoblot with the anti WWP1 antibody on extracts from 293T cells expressing WWP1, 293T and HeLa cells. (B) Blot was performed with the anti-WWP1 antibody on lysates of 293T cells transfected with scramble siRNA (si scr) or a pool of siRNA targeting WWP1 (si WWP1). Tubulin serves as loading control. (C) Membrane and cytosolic fractions of 293T cells were blotted with ezrin and WWP1 antibodies. The receptor Met was used as a control for cell fractionation. (D) Immunofluorescence was performed on LLC-PK1 cells stably expressing WWP1-GFP with an anti- E-cadherin (red) or anti ezrin (red) antibodies and visualized by wide-field 3D sectioning microscope with Z step every 0.2 µm. Two different focal planes are shown. Scale bar: 10 µm.

To address in which cellular compartment the ezrin/WWP1 complex could form we performed membrane/cytosol fractionation of 293T cell lysates. We developed an antibody to human WWP1 against a peptide comprising amino acids 163–176 that recognized one band only in human cell lines with the molecular weight of WWP1 (Fig. 3A). A complete disappearance of the band corresponding to WWP1 was observed when 293T cells were treated with siRNA targeting WWP1 further confirming the specificity of our antibody (Fig. 3B). As previously reported, ezrin is present both in the cytoplasm and associated with the membranes. However, because a small pool of ezrin (∼10%) is associated with the membrane [3], it can be detected in this fraction only when it is concentrated. In contrast, WWP1 is mostly present in the membrane fraction with detectable levels in the cytoplasmic fraction (Fig. 3C). We then performed immunofluorescence analysis to determine in which cellular compartment WWP1 was present. It has been previously shown that WWP1 localizes to the nucleus and cytoplasm in MDCK and HeLa cells [17], [18]. By immunofluorescence the antibody we developed to human WWP1 did not recognize the endogenous porcine protein. We therefore generated pools of LLC-PK1 cell stably expressing GFP-tagged forms of WWP1. LLC-PK1 cells are derived from kidney proximal tubules and display characteristic features of polarized absorptive cells. In these cells, WWP1 showed colocalization with endogenous ezrin at the microvilli and with E-cadherin at the cell-cell junctions (Fig. 3D).

### Ezrin is Ubiquitylated by WWP1

**Figure 4:**
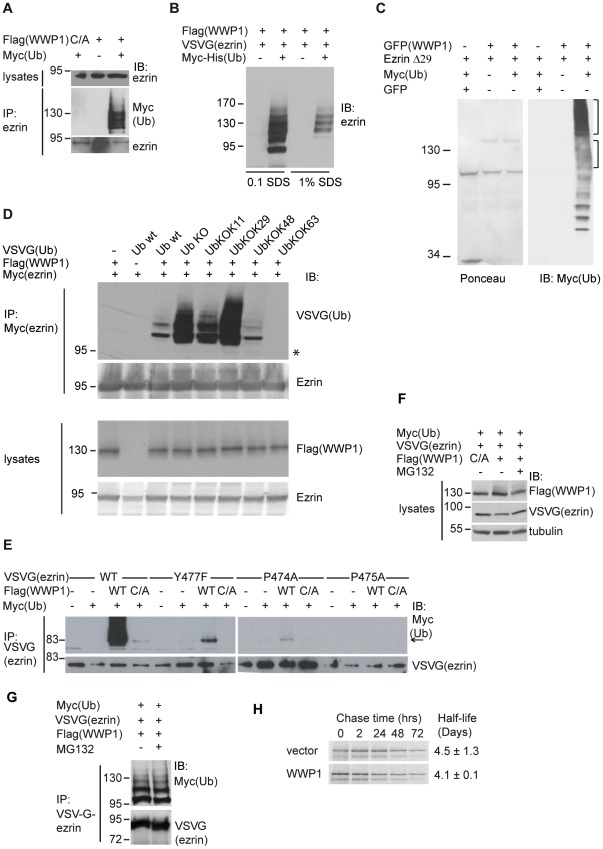
Ezrin ubiquitylation by WWP1. (A) Lysates from 293T cells transfected with the indicated combinations of wild type flag-WWP1, flag-WWP1 C890A (C/A) and Myc-ubiquitin were immunoprecipitated with an anti-ezrin antibody followed by a blot with a Myc antibody. (B) 293T cells expressing Flag-WWP1, ezrin-VSVG and Myc-His-ubiquitin were lysed either in RIPA buffer or RIPA buffer containing 1% SDS and the ubiquitylated proteins were purified on TALON beads. The immunoblot was performed with an anti-VSVG antibody. (C) In vitro ubiquitylation was performed in presence of the indicated proteins. Left panel shows a Ponceau staining. Right panel: the blot was performed with an anti-Myc antibody to detect ubiquitylated protein. The brackets indicate the extent ubiquitylation of ezrin Δ29 and WWP1 (D) Ezrin is multi-ubiquitylated. 293T cells were transfected with the indicated plasmids. Upper panels: Immunoprecipitation of ezrin-Myc was followed by immunoblotswith the anti-VSVG (ubiquitin) and anti-ezrin antibodies. The asterisk indicates the position of non ubiquitylated ezrin. Lower panels: the immunoblots were performed on cell lysates with anti-Flag (WWP1) and anti-ezrin antibodies. (E) Ezrin ubiquitylation requires the PPXY motif. Lysates from 293T cells transfected with the indicated combinations of wild type flag-WWP1, flag-WWP1 C890A, wild type ezrin-VSVG, P474A, P475A, Y477F ezrin-VSVG and Myc-ubiquitin were immunoprecipitated with an anti-ezrin antibody followed by immunoblot with anti-Myc and -VSVG antibodies. (F) Ezrin steady state levels are not affected by WWP1 expression. 293T cells were transfected with the indicated combinations of plasmids and treated or not with the proteasomal inhibitor MG132. Immunoblots were performed with the indicated antibodies. Tubulin serves as a loading control. (G) Ezrin ubiquitylation with MG132 treatment. 293T cells were transfected with the indicated plasmids and treated or not with MG132. Immunoprecipitation with anti-VSVG antibody was followed by immunoblotting with anti-Myc and -VSVG antibodies. (H) Expression of WWP1 does not affect the half-life of ezrin. Stable Flp-In™ T-REx™ 293 cell lines expressing wild type PC- tagged WWP1 or vector alone were pulse-labeled for 15 min with ^35S^methionine/cysteine and chased for 0, 2, 24, 48, and 72 hours. Autoradiography of ezrin immunoprecipitates is shown.

Given the interaction between WWP1 and ezrin, we tested whether WWP1 induced the ubiquitylation of ezrin. We expressed WWP1 wild type or catalytically inactive (C883A) forms in 293T cells in the presence of ubiquitin followed by immunoprecipitation of endogenous ezrin. Expression of WWP1, but not WWP1 (C883A), led to a distinct pattern of high molecular weight bands that correspond to multi−/poly-ubiquitylated ezrin (Fig. 4A). This pattern of ubiquitylation was still observed when denaturing conditions were used to purify ubiquitylated conjugates (Fig. 4B). We next tested whether WWP1 promoted ezrin ubiquitylation in vitro. We performed an ubiquitin conjugation assay using purified GFP-WWP1 bound to beads and purified GST-ezrin in presence of ubiquitylation reagents. No ezrin ubiquitylation could be observed with the full length protein (unpublished data). One possible reason for this negative result would be that the full length ezrin is in a closed conformation therefore its interaction with WWP1 might be hampered resulting in a reduced accessibility to the ubiquitylation site. We therefore used an open ezrin form in which the last 29 amino acids were deleted (ezrin Δ29). As shown in figure 4C, a ladder of ubiquitylated ezrin was observed. We also observed a ladder of ubiquitin intensifying above the molecular weight band for WWP1 suggesting that WWP1 may self-ubiquitylate. Under the same conditions, no ladder was observed when WWP1 and ezrin Δ29 were incubated in absence of ubiquitin or when WWP1 was replaced by GFP alone (Fig. 4C).

To identify the type of isopeptide linkage catalyzed by WWP1, we coexpressed ezrin-Myc, WWP1 and VSVG-tagged ubiquitin either wild type, mutated in all lysine residues used to polymerize ubiquitin residues (UbKO) or in all but one lysine residue (Ub KOK11, KOK29, KOK48, KOK63). The same pattern of ubiquitylation was observed with the different forms of ubiquitin. The expression level of ubiquitin wild type observed in total cell lysates was lower (data not shown) resulting in lower intensity of ezrin ubiquitylated bands. Moreover, Ub KOK63 is poorly incorporated (Brou, C. personnal communication) therefore the ezrin ubiquitylated bands could only be detected after longer exposure (unpublished results) (Fig. 4D). Notably, cells expressing ubiquitin KO, which can only form isopeptide bond between the last glycine (G76) and lysine residues in ezrin, displayed the same profile of ubiquitylation as cells expressing wild type ubiquitin. Altogether these data provided compelling evidence that ezrin is monoubiquitylated by WWP1 at more than one site since several bands could be detected.

We also found that the PPVY^477^ motif of ezrin was required for this ubiquitylation. When compared to wild-type ezrin, this pattern of multi-ubiquitylation was not present in cells expressing ezrin Y477F, P474A or P475A (Fig. 4E). In ezrin Y477F and P474A mutants, but not in ezrin P475A mutant, one band was present, corresponding to a shift of 8 Kd, the size of one ubiquitin molecule. Since this band was also present in the extract of cells expressing the catalytically inactive form of WWP1 it suggests that the monoubiquitylation is not dependent on the activity of WWP1. All together, these results indicate that ezrin multi-ubiquitylation by WWP1 requires the interaction between these two proteins via the PPVY^477^motif in ezrin.

As WWP1-dependent ubiquitylation has been shown to affect the stability of several of its substrates, we determined the possible role of WWP1 in the degradation of ezrin. Overexpression of WWP1 in 293T cells failed to cause any changes in ezrin expression levels (Fig. 4F). Consistent with this conclusion is the observation that the addition of the proteasomal inhibitor MG132 had no effects on the total amount of ezrin present in the cells (Fig. 4F), nor on its level of ubiquitylation (Fig. 4G). To further confirm that WWP1 does not regulate the stability of ezrin, WWP1 was stably expressed in Flp-InTM T-RExTM 293 cells. Pulse chase experiments were performed with cells transfected either with the vector alone or with the vector expressing WWP1. We found that the rates of endogenous ezrin degradation were similar between the WWP1 expressing cell line and cells transfected with the vector alone. We found the ezrin half life to be 4.5+/−1.3 days for vector and 4.07+/−0.1 for WWP1 expressing cells (Fig. 4H). Therefore, ubiquitylation of ezrin by WWP1 is involved in functions other than the stabilization of the protein.

### WWP1 Overexpression Upregulates Met

**Figure 5:**
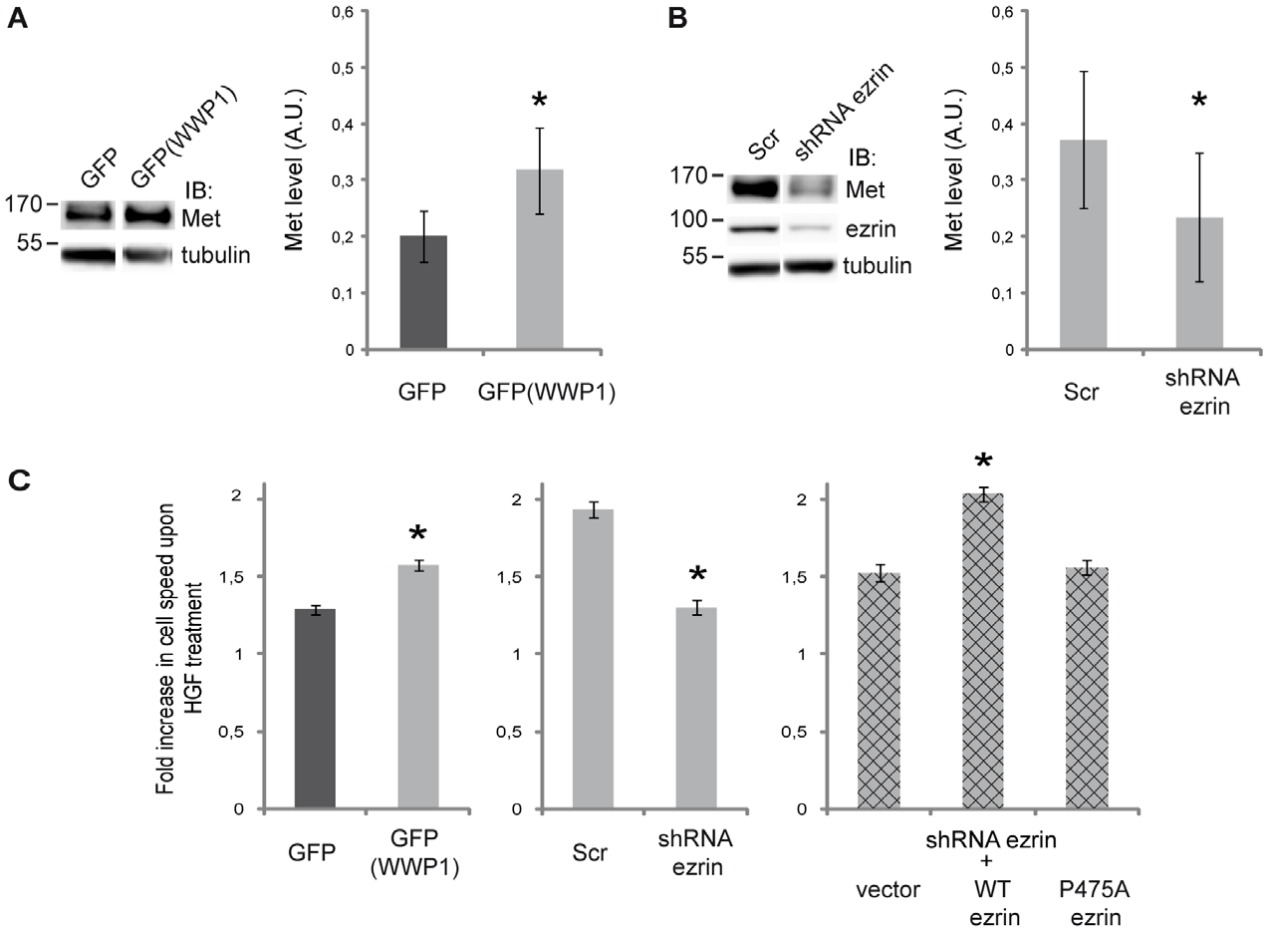
Ezrin ubiquitylation by WWP1 is required for Met upregulation. (A) Met levels in LLC-PK1 cells expressing GFP-WWP1 or GFP alone. Left panel: Blots were performed with anti-Met and anti-tubulin antibodies. Right panel: The quantification of Met level normalized to tubulin results from five independent experiments. Data are expressed as mean ± SEM, *P<0.05 (paired t-test). (B) Ezrin depletion prevents Met upregulation. Stable GFP-WWP1 cells were transfected with plasmids coding for shRNA targeting ezrin (shEz) or a scramble sequence (Scr). Left panel: Cell lysates were blotted with the indicated antibodies. Right panel: the quantification of Met level normalized to tubulin results from five independent experiments. Data are expressed as mean ± SEM, *P<0.05 (paired t-test). (C) Cell speed measured in a wound healing assay. In each graph, the bars represent the increase in the speed of cells treated with HGF as compared to the speed of untreated cells. Left panel: LLC-PK1 cells expressing either GFP or GFP-WWP1. Middle panel: LLC-PK1 cells expressing GFP-WWP1 and transfected with plasmids coding for scramble shRNA (Scr) or shRNA targeting ezrin. Right panel: LLC-PK1 cells expressing GFP-WWP1 and shRNA targeting ezrin were transfected with either an empty vector (vector) or plasmids coding for ezrin wild type or ezrin P475A. The data correspond to three independent experiments. Significance was tested using a two-way ANOVA model with interaction (SigmaStat). The pairwise comparisons were performed with the Student-Newman-Keuls method using an α risk of 0.05.

WWP1 is involved in the positive or negative regulation of several receptor targets [17]–[19], [27]. We previously showed that ezrin plays a role in HGF-induced cell morphogenesis, motility [4] and scattering [9]. Therefore we decided to investigate whether the interaction between ezrin and WWP1 might play a role in regulating the stability and/or the activity of Met. We examined Met protein levels in LLC-PK1 cells stably expressing GFP-WWP1. We found that after 5 h starvation and in the absence of HGF stimulation, the level of Met was upregulated in cells expressing WWP1 when compared to cells overexpressing the GFP alone (Fig. 5A). To determine whether this effect was dependent on ezrin, we knocked-down ezrin by transiently transfecting LLC-PK1 cells, overexpressing WWP1, with a plasmid encoding short hairpin RNA targeting ezrin (shEz). Expression of the shRNA led to an efficient knock down of ezrin (Figure 5B). In cells expressing GFP-WWP1 and the shRNA for ezrin we observed a decrease in Met levels compared to cells transfected with GFP alone. Altogether, these results indicated that WWP1 and ezrin cooperate in the control of Met levels.

To determine if the expression of WWP1 had an effect on the activity of the receptor, we measured the cell response to HGF during wound healing. We measured the speed of LLC-PK1 cells stably expressing either GFP-WWP1 or GFP in presence or in absence of HGF during wound healing. Upon HGF treatment the speed of the cells was increased when compared to that of unstimulated cells. This increase was higher in cells expressing GFP-WWP1 when compared to cells expressing GFP alone (Figure 5C left). To determine if this increased response of Met in the presence of WWP1 was dependent on ezrin we performed the wound healing on cells treated either with scramble siRNA as control or siRNA targeting ezrin. Cells depleted for ezrin showed reduced response to HGF stimulation when compared to cells transfected with scramble siRNA (Figure 5C, middle panel). We next seeked to determine if the increase in HGF response we observed required ubiquitylated ezrin. We transfected cells expressing GFP-WWP1 and depleted for ezrin either with empty vector, wild type ezrin or with ezrin P475A that cannot be multi- ubiquitylated and we monitored the speed of the cells in the wound healing assay. Whereas the overexpression of wild type ezrin restored HGF response to level comparable to that of cells transfected with non targeting sequences (Scr), cells expressing ezrin P475A show speed levels similar to that of controls i.e. silenced cells transfected with an empty plasmid (vector). Altogether these data indicate that the ubiquitylation of ezrin by WWP1 is required for WWP1-dependent increase in Met activity.

## Discussion

We and others have previously shown that ezrin participates in several HGF-induced cellular responses downstream of Met signalling. Here we show that ezrin, through its ubiquitylation by WWP1, also act upstream by controlling the level of the receptor.

WWP1 binds to two domains of ezrin: the FERM domain and the PPVY^477^ motif present in its C-ter domain. The interaction between the PPVY^477^ motif in ezrin and the WW domains of WWP1 is a key step since mutation of any amino acids in this motif abolishes ezrin ubiquitylation although an interaction is still observed between the two proteins. For these reasons it is not possible to establish a firm conclusion on whether Y477 phosphorylation is an absolute requirement for the interaction of ezrin with WWP1, although WWP1 has been isolated in a phospho-dependent screen. Our results suggest that the interaction of the PPVY^477^ motif with the WW domains may also play a role in the activation of WWP1. Indeed, several HECT domain-containing E3 ligases are self-inhibited and can be activated by different means including the binding of their WW domains to the PPXY motif of their binding partners [28]–[30].

We have previously described an interaction between the Fes kinase SH2 domain and ezrin that occurs through phosphorylated tyrosine 477 of ezrin [9]. Interestingly, ezrin/Fes interaction at cell–cell contacts plays an essential role in HGF-induced cell scattering Therefore two different proteins which regulate Met receptor activity bind to this linker region through their SH2 and WW domains respectively. This raises the question of whether the binding of these two proteins is exclusive or whether it can occur simultaneously with different pools of ezrin consistent with a role for ezrin in organizing signalling complexes.

We report here, for the first time that ezrin is ubiquitylated by WWP1. We did not observe changes in the half-life of ezrin when the cells express WWP1 suggesting that ezrin ubiquitylation by WWP1 serves functions other than degradation of ezrin. We show here that the interaction of ezrin with WWP1 upregulates the Met receptor in the absence of HGF stimulation. Various mechanisms have been shown to regulate Met stability and degradation. One mechanism involves c-Cbl ubiquitin ligase. The c-Cbl-mediated ubiquitylation of Met following HGF stimulation triggers Met internalization and down regulation. Uncoupling Met from c-Cbl-mediated ubiquitylation increases the stability of the receptor that acquires transforming activity in fibroblast and epithelial cells [31]–[33]. Moreover, it has been reported that the transmembrane leucine-rich repeat protein LRIG1 interacts with and destabilizes Met in the absence of HGF and in a Cbl-independent manner through a mechanism that is not as yet defined [34]. The effect of WWP1 expression on Met stability that we describe here has previously been observed for EGFR and ErbB2. It has been shown that WWP1 upregulates these receptors by inhibiting the activity of the RING finger protein 11 (RNF11) E3 ubiquitin ligase that targets EGFR and ErbB2 for degradation [19]. We propose that a similar mechanism may operate for Met regulation. WWP1 may regulate Met stability and consequently its activity indirectly by targeting positive or negative regulators of the receptor. In this context, ubiquitylated ezrin may function as an adaptor recruiting the specific substrates for WWP1.

Our results demonstrate that in a wound healing assay, expression of WWP1 leads to higher migratory rates in response to HGF stimulation. A likely explanation is that the upregulation of Met observed in cells expressing GFP-WWP1 better sustains signalling upon HGF stimulation. Both the upregulation of the receptor and its increased signalling function observed in the process of cell migration also require ubiquitylated ezrin. We propose that, through its ubiquitylation, ezrin may facilitate the assembly of a complex comprising WWP1 to consequently stabilize the receptor.

Genetic and functional analyses in human cancers have shown that WWP1 is a potential oncogene. Genomic amplification of WWP1 has been detected in approximately 40% of breast and prostate human cancers [35], [36]. Knockdown of WWP1 in breast cancer epithelial cell lines induces growth arrest and apoptosis further supporting a role in tumorigenesis [36]. Recent findings indicate that ezrin is required for metastasis of breast carcinoma, osteosarcoma and HGF-induced rhabdomyosarcoma [37]–[39]. Our results link WWP1 and ezrin to the Met receptor activity, providing a molecular basis to the role of ezrin and WWP1 in tumor progression. It would be thus important to address the role of ezrin/WWP1 interaction in the regulation of Met activity in tumor cells and in metastasis as Met plays a central role in these processes [1].

## Acknowledgments

The authors are grateful to Dr. Christel Brou (Institut Pasteur) for the gift of the ubiquitin plasmids and for helpful discussion. They greatly acknowledge the Nikon Imaging Centre at the Institut Curie-CNRS and Vincent Fraisier for image deconvolution. They thank all Hybrigenics staff for help with yeast two-hybrid analysis.
